# Case Report: CYLD cutaneous syndrome with malignant transformation to spiradenocarcinoma: cooperative effects of CYLD truncation and an MSH2 clamp-domain variant in an Ecuadorian patient

**DOI:** 10.3389/fmed.2026.1703885

**Published:** 2026-02-18

**Authors:** Carlos Reyes-Silva, Gabriela Jaramillo-Koupermann, Maritza Quishpe, Rosa Pacheco, Skehirly Burgos-Tapia, Ana Karina Zambrano, Alejandro Cabrera-Andrade

**Affiliations:** 1 Unidad de Genética, Hospital de Especialidades Eugenio Espejo, Quito, Ecuador; 2 Laboratorio de Biología Molecular, Subproceso de Anatomía Patológica, Hospital de Especialidades Eugenio Espejo, Quito, Ecuador; 3 Anatomía Patológica, Hospital de Especialidades Eugenio Espejo, Quito, Ecuador; 4 Escuela de Enfermería, Facultad de Ciencias de la Salud, Universidad de Las Américas, Quito, Ecuador; 5 Facultad de Ciencias de la Salud Eugenio Espejo, Centro de Investigación Genética y Genómica, Universidad UTE, Quito, Ecuador; 6 Grupo de Bio-Quimioinformática, Universidad de Las Américas, Quito, Ecuador

**Keywords:** CYLD cutaneous syndrome, CYLD, dermatologic neoplasm, Ecuador, mismatch repair, MSH2, rare genetic disorders, spiradenocarcinoma

## Abstract

**Background:**

CYLD cutaneous syndrome (CCS) is a rare autosomal dominant disorder caused by germline *CYLD* variants and characterized by multiple skin adnexal tumors. Malignant transformation is uncommon, and cooperative genetic events remain poorly defined, particularly in underrepresented populations.

**Case presentation:**

We report a 61-year-old Ecuadorian woman with multiple scalp cylindromas and spiradenomas, including one spiradenocarcinoma. Family history was notable for malignancies in first- and second-degree relatives. Whole-exome sequencing identified a heterozygous nonsense *CYLD* variant (c.1207C > T; p.Gln403Ter), classified as likely pathogenic, and a homozygous missense *MSH2* variant (c.1609A > G; p.Lys537Glu) of uncertain significance. Histopathology confirmed malignant transformation, while immunohistochemistry showed preserved MSH2 expression with a microsatellite-stable phenotype. Nevertheless, a functional impact of the *MSH2* variant cannot be excluded. Consistent with these observations, *in silico* modeling demonstrated that *CYLD* truncation eliminates the catalytic USP domain and regulatory motifs, abolishing deubiquitinase activity, whereas the *MSH2* substitution affects a conserved residue in the clamp domain, likely destabilizing the MSH2–MSH6 complex despite intact nuclear localization.

**Conclusion:**

This is the first genetically confirmed case of CCS in Ecuador and among the few reported in South America. Beyond expanding the geographic spectrum, our findings highlight the value of integrating genomic and protein analyses to uncover cooperative mechanisms of malignant progression. Such integrative genomic approaches refine diagnosis, enhance genotype–phenotype interpretation, and deepen understanding of malignant transformation in CCS, particularly in underrepresented populations.

## 1 Introduction

CYLD cutaneous syndrome (CCS) is a rare autosomal dominant disorder characterized by the development of multiple benign skin adnexal tumors, most commonly affecting the scalp, face, and neck. Despite its clinical relevance, CCS remains underreported, with most cases described in Europe, North America, and parts of Asia (1), and only isolated reports from South America (2–4).

CCS encompasses a spectrum of phenotypes historically described as Brooke-Spiegler syndrome, familial cylindromatosis, and multiple familial trichoepithelioma, now recognized as variable manifestations of a single condition caused by pathogenic *CYLD* variants (5). *CYLD*, located on chromosome 16q12.1, encodes a cytoplasmic tumor suppressor protein with deubiquitinase activity that regulates key signaling pathways, including NF-κB, Wnt, Notch, and TGF-β. CYLD exerts its tumor suppressor function primarily by removing K63- and M1-linked ubiquitin chains from central adaptor proteins (6). Loss-of-function variants, often nonsense or frameshift changes upstream of the ubiquitin-specific protease (USP) domain, abolish deubiquitinase activity. Tumorigenesis follows a two-hit model, with germline heterozygosity complemented by somatic inactivation of the wild-type allele (7, 8).

Although CCS is predominantly characterized by benign adnexal tumors, malignant transformation is a recognized but rare event. The true frequency of malignant transformation remains uncertain, as CCS is rare and many benign lesions are excised before progression can be assessed. Previous reports have documented spiradenocarcinomas, cylindrocarcinomas, basal cell adenocarcinoma–like carcinomas, and cutaneous squamous cell carcinoma arising from long-standing spiradenomas or cylindromas, often through *in situ* progression (1, 2, 9–11). Low-grade basal cell adenocarcinoma–like tumors usually follow an indolent course, whereas high-grade spiradenocarcinomas and invasive adenocarcinomas behave aggressively, with potential for local invasion and distant metastasis (2, 9, 12). Integrative molecular analyses of CYLD-mutated tumors further indicate that malignant progression is often accompanied by additional somatic alterations, including *TP53* mutations in a subset of high-grade neoplasms and recurrent changes in epigenetic regulators such as *EP300* and *DNMT3A* in advanced lesions (12, 13). Notably, these cooperative events have predominantly involved chromatin-modifying genes, developmental signaling pathways, and classic tumor suppressors in malignant spiradenomas and cylindromas, whereas central mismatch repair (MMR) genes have not typically been implicated in CCS-related tumorigenesis.

Here, we report the first genetically confirmed case of CCS in Ecuador. A 61-year-old woman presented with multiple scalp adnexal tumors, including cylindromas, spiradenomas, and a spiradenocarcinoma. Her family history included cancer and adnexal tumors in first-degree relatives. Genetic testing identified a heterozygous nonsense variant in *CYLD* (c.1207C > T; p.Gln403Ter), absent from population databases but previously reported in a CCS cohort, and a homozygous missense variant in *MSH2* (c.1609A > G; p.Lys537Glu). A genetic ancestry analysis was performed to contextualize these findings within the genetic background of Latin America, where populations remain underrepresented. This case expands the geographic and mutational spectrum of CCS and raises the possibility of cooperative germline alterations that contribute to malignant progression.

## 2 Case description

### 2.1 Patient presentation and clinical assessment

**Figure 1:**
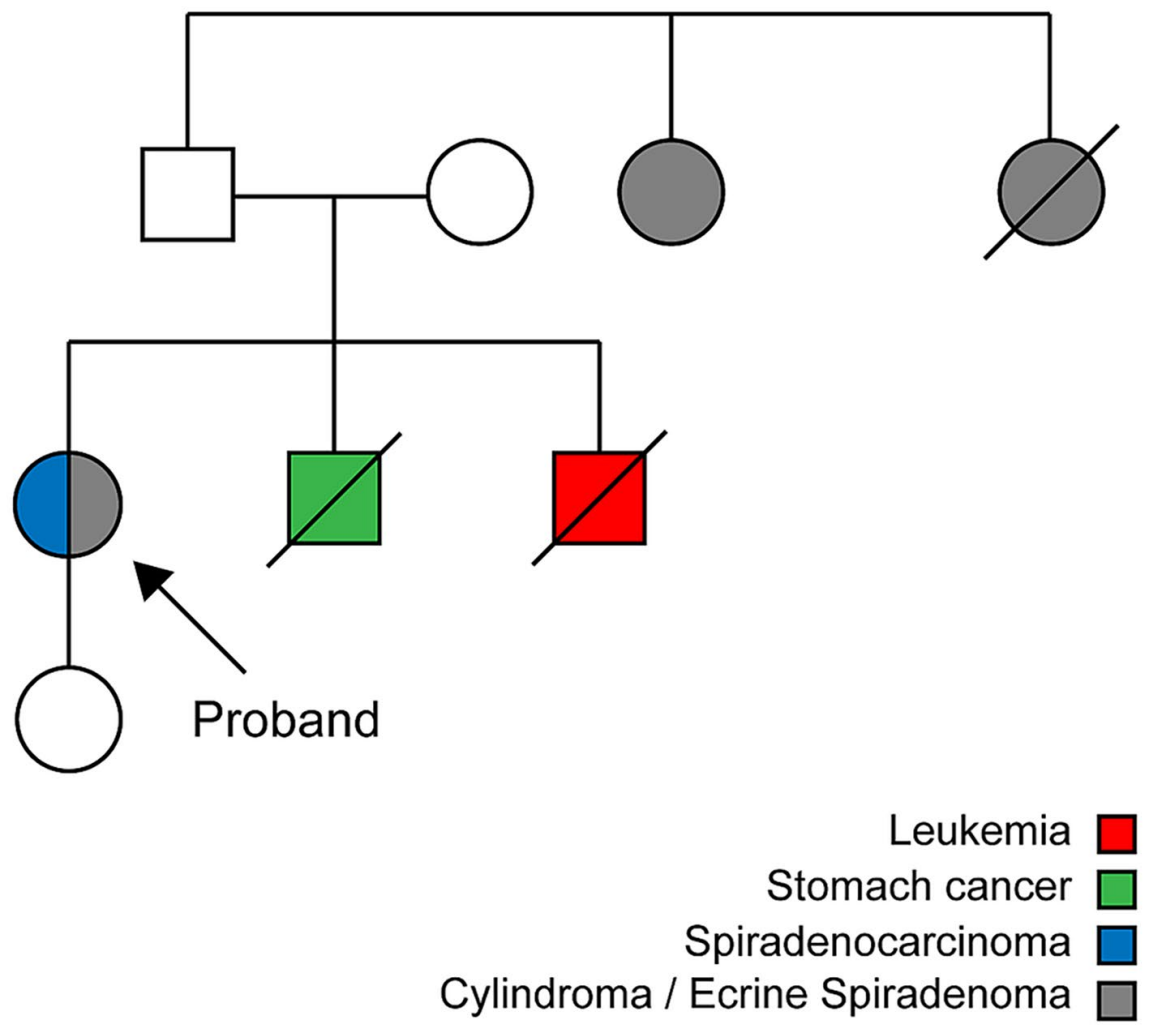
Pedigree of the proband with CYLD cutaneous syndrome. The proband (arrow) presented with multiple cylindromas, spiradenomas, and a spiradenocarcinoma. Affected relatives and cancer diagnoses are indicated by color coding as shown in the legend.

A 61-year-old woman from Quito presented with multiple nodular lesions on the scalp and face. The first scalp lesion appeared at age 18 as a 5 cm nodule in the frontal region and was excised at age 26. Additional nodules developed in the right parietal region at age 29 and in both parietal areas at age 35, all of which were surgically removed. At age 50, new retroauricular lesions measuring approximately 4 cm were excised, and she has since remained under periodic oncologic follow-up. The patient’s medical history included well-controlled hypertension and type 2 diabetes mellitus treated with chlorthalidone, enalapril, and metformin. Routine laboratory tests showed mild hyperglycemia (glucose 145 mg/dL) and hypercholesterolemia (total cholesterol 269 mg/dL), with otherwise normal hematologic and biochemical parameters. The initial differential diagnosis included squamous cell carcinoma, but the multiplicity and distribution of adnexal tumors suggested CCS. Her family history included gastric cancer in one brother (70 years old), leukemia in another brother (80 years old), and facial tumors in two paternal aunts diagnosed after 65 years of age (Figure 1). Overall, the clinical course spanned more than three decades, with recurrent scalp adnexal tumors appearing from early adulthood to her sixth decade of life.

### 2.2 Histopathology and immunohistochemical analysis

**Figure 2:**
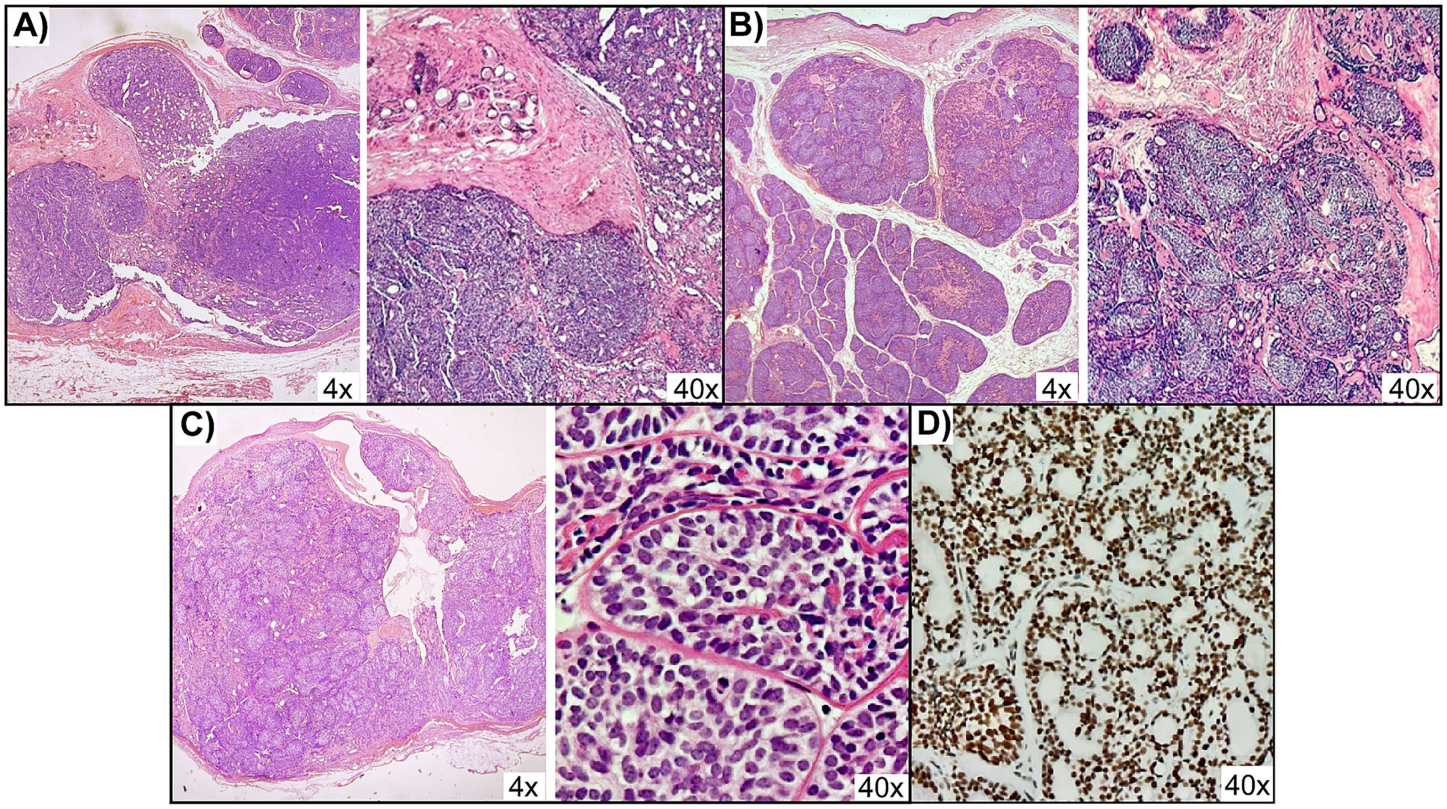
Histopathology and immunohistochemistry of scalp adnexal tumors. Hematoxylin and eosin (H&E) staining showing panoramic 4× views with corresponding 40× details of **(A)** cylindroma with nests of basaloid cells surrounded by a thick hyalinized basement membrane, **(B)** eccrine spiradenoma with well-defined basaloid cell aggregates in a hyalinized stroma, and **(C)** spiradenocarcinoma with nuclear atypia, hyperchromasia, and increased mitotic activity. **(D)** Immunohistochemistry for MSH2 in the spiradenocarcinoma lesion, showing preserved nuclear expression.

The excised scalp nodules were dome-shaped, firm, and skin-colored to pink, ranging from 0.5 to 4 cm in diameter, with some coalescing into larger plaques. At low power, the lesions formed well-circumscribed, dome-shaped silhouettes that mirrored the clinical appearance of the scalp nodules (Figures 2A–C). Histopathological examination revealed multiple benign adnexal tumors consistent with cylindromas and eccrine spiradenomas. Cylindromas displayed nests of basaloid cells arranged in a jigsaw-like pattern, surrounded by a thick hyalinized basement membrane (Figure 2A), whereas eccrine spiradenomas showed well-defined basaloid cell aggregates in a hyalinized stroma (Figure 2B). A separate lesion showed an expansile growth pattern with focal infiltration into the adjacent dermis and displayed malignant cytologic features, including nuclear enlargement, hyperchromasia, and an elevated mitotic rate of up to seven mitoses per ten high-power fields (Figure 2C), confirming the diagnosis of spiradenocarcinoma. Overall, the histologic profile supported a diagnosis of CCS with secondary malignant transformation of an adnexal tumor. Immunohistochemistry (IHC) showed preserved nuclear expression of MSH2, and microsatellite testing confirmed a microsatellite-stable (MSS) phenotype (Figure 2D).

### 2.3 Genetic findings

Whole-exome sequencing was performed on genomic DNA from the patient using the Twist Bioscience Exome V2.0 capture and PacBio Onso short-read platform (SBB™ chemistry), achieving a mean coverage of ≥100×. Reads were aligned to the GRCh38 reference genome, and variant calling and annotation were conducted with the Bitgenia pipeline and B-platform v3.1.7. The analysis identified a heterozygous nonsense variant in *CYLD* (c.1207C > T, NM_015247.3), classified as likely pathogenic according to ACMG/AMP criteria (PVS1, PM2). Additionally, a homozygous missense variant in *MSH2* (c.1609A > G, NM_000251.3) was detected. This variant is absent from ClinVar, gnomAD, and LOVD and was classified as a variant of uncertain significance (VUS).

Sanger sequencing confirmed both variants and enabled a segregation analysis. The *CYLD* variant was present in heterozygosity in the patient and her daughter, while the *MSH2* variant was homozygous in the patient and heterozygous in the daughter, consistent with germline inheritance. No additional family members were available for genetic or histopathological evaluation. Primers for *CYLD* c.1207C > T (NG_012061.1) and *MSH2* c.1609A > G (NG_007110.2) were designed using Primer-BLAST^1^ and are listed in Supplementary Table S1.

To contextualize these findings, ancestry proportions were estimated using 46 ancestry-informative markers genotyped by multiplex PCR and capillary electrophoresis. The patient’s ancestry profile was: 39.6% Native American, 42.2% European, and 18.2% African. Details are shown in Supplementary Figure S1.

### 2.4 *In silico* structural and functional analysis

**Figure 3:**
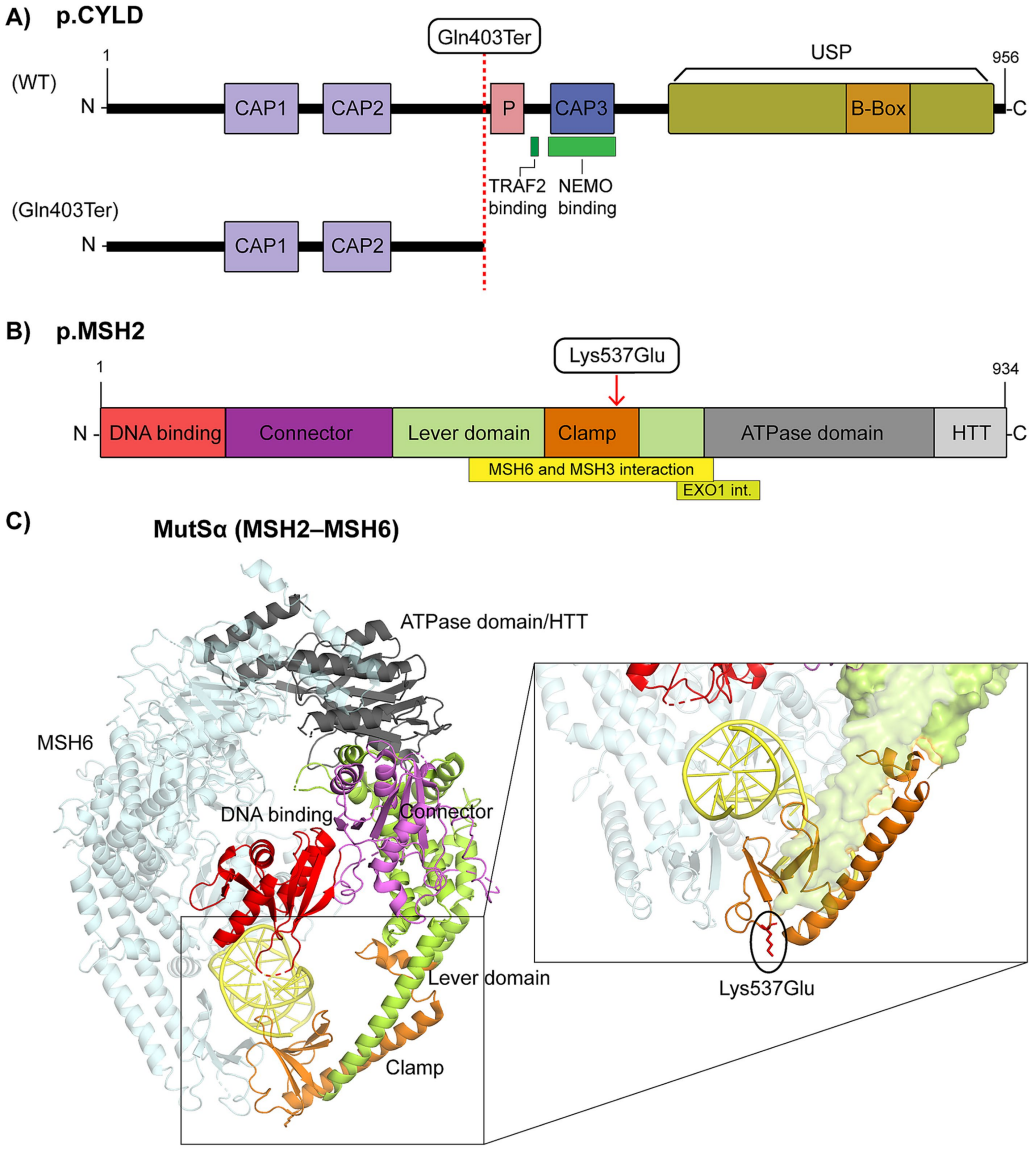
Structural impact of *CYLD* and *MSH2* variants. **(A)** Schematic representation of the CYLD protein (NP_056062.1) showing three CAP-Gly domains, phosphorylation site (P), ubiquitin-specific protease (USP) domain, and B-box, with annotated TRAF2 and NEMO binding sites. The truncated protein resulting from the p.Gln403Ter variant is shown below. **(B)** Domain architecture of MSH2 (NP_000242.1), including the DNA-binding, connector, lever, clamp, ATPase, and helix-turn-helix (HTH) domains, with MSH6/MSH3 and EXO1 interaction sites. The position of the p.Lys537Glu variant is indicated. **(C)** Three-dimensional structure of human MutSα (MSH2–MSH6) bound to DNA (PDB: 2O8C), with MSH2 domains colored as in **(B)**. The inset highlights the lever and clamp region, indicating the position of the Lys537Glu variant.

The annotation of the CYLD protein using UniProt (Q9NQC7), InterPro,^2^ SMART,^3^ and Pfam identified three CAP-Gly domains, a serine phosphorylation cluster, TRAF2/NEMO interaction sites, the B-box subdomain, and the C-terminal USP catalytic domain. The c.1207C > T variant introduces a premature stop codon upstream of the USP domain, leading to truncation of CAP-Gly 3, the TRAF2/PVQES motif, the phosphorylation cluster, and the USP catalytic core (Figure 3A; Supplementary Table S2).

MSH2 annotation (UniProt P43246) showed DNA-binding, connector, lever, clamp, ATPase, and HTH domains, plus interaction sites with MSH6/MSH3 and EXO1 (Figure 3B). The p.Lys537Glu variant maps to the clamp domain, critical for DNA binding and heterodimer stabilization. To evaluate the potential impact of this variant, we applied a multilevel *in silico* approach that assessed evolutionary conservation, structural context, and predicted pathogenicity.

The conservation analysis with PhyloP and PhastCons demonstrated that Lys537 is highly conserved, indicating functional constraint at this site. Multiple *in silico* predictors classified the substitution as possibly damaging: SIFT (score = 0), PolyPhen-2 (possibly damaging, score ≈ 0.76), and AlphaMissense (pathogenicity score = 0.6059). Structural stability predictions with FoldX and DUET indicated a destabilizing effect, consistent with reduced protein stability. The residue-level analysis with MISTIC2, based on the crystallographic structure of the clamp domain (PDB 2O8B), confirmed that Lys537 lies within a structurally constrained region. Substitution with glutamate disrupts local interaction networks essential for mismatch recognition and complex stability. Mapping onto the human MutSα heterodimer (MSH2–MSH6, PDB 2O8C) demonstrated that this residue is positioned at the interface of the clamp and lever domains, in proximity to the DNA-binding groove (Figure 3C).

### 2.5 Follow-up and clinical management

The patient underwent a complete surgical excision of symptomatic scalp tumors, including the lesion diagnosed as spiradenocarcinoma. Postoperative recovery was uneventful. Given the risk of recurrence and malignant transformation inherent to CCS, she was referred for multidisciplinary follow-up with dermatology and oncology. A surveillance plan was established, consisting of a full dermatologic examination every 6 months and annual imaging of the scalp and cervical lymph nodes.

Genetic counseling was provided to the patient and her family. Her daughter, who carries the heterozygous *CYLD* variant, was informed of her increased risk of developing cutaneous adnexal tumors and referred for baseline dermatological evaluation. Extended family members were advised of the availability of predictive testing.

## 3 Discussion

We describe a patient with CCS and malignant transformation to spiradenocarcinoma, carrying two rare germline variants: *CYLD* c.1207C > T (p.Gln403Ter, heterozygous) and *MSH2* c.1609A > G (p.Lys537Glu, homozygous). Both were absent from ClinVar, gnomAD, and LOVD, meeting PM2 for rarity; the *CYLD* variant additionally fulfilled PVS1 and was classified as likely pathogenic, while the *MSH2* variant remains a VUS (14). Histopathologic examination of our case revealed multiple scalp-limited adnexal tumors, including cylindromas and eccrine spiradenomas, as well as a single spiradenocarcinoma exhibiting nuclear atypia, hyperchromasia, and increased mitotic activity. This spectrum of lesions is characteristic of CCS and, although malignant progression is rare, supports a diagnosis of secondary malignant transformation from a benign precursor (15). Notably, p.Gln403Ter has previously been described in a patient with a benign scalp cylindroma and no evidence of malignant transformation (16), suggesting that this variant alone may not be sufficient to drive malignant progression.

Mechanistically, truncating *CYLD* variants, particularly between exons 8 and 20, support the existence of a mutational hotspot associated with CCS. These variants typically disrupt the USP domain, resulting in the loss of deubiquitinase activity and tumor suppressor function. The nonsense variant identified in our patient is consistent with this pattern (17, 18), likely reflecting convergent pathogenetic mechanisms rather than a shared founder origin. The p.Gln403Ter variant introduces a premature stop codon upstream of the USP domain, resulting in a complete loss of deubiquitinase activity. It also removes regions for substrate recognition (CAP-Gly 3 with NEMO site, TRAF2/PVQES motif), subcellular localization (B-box, CAP-Gly 3), and structural stability (USP domain), as well as the regulatory phosphorylation cluster, disrupting NF-κB targeting, cytoplasmic retention, and modulation of catalytic activity (19, 20). Similar to other truncations occurring upstream of the USP domain, this variant likely produces an unstable protein with no regulatory effect on NF-κB signaling. Accordingly, the absence of these domains is expected to impair CYLD’s ability to modulate key pathways such as NF-κB, Wnt/β-catenin, TGF-β, and MAPK (21–23).

Phenotype–genotype correlations have shown that nonsense variants are more frequently associated with disease manifestations confined to the head and neck region (24). Studies of patients with cylindroma and spiradenoma histologies have also reported a high prevalence of truncating variants, particularly nonsense and frameshift (24–26). Although statistically significant associations between specific variant types and tumor histologies have not been formally demonstrated, these trends suggest that both the nature and position of the *CYLD* variant may influence the anatomical distribution and clinical expression of CCS. Recent reports have likewise described *CYLD* variants leading predominantly to multiple facial trichoepitheliomas (27), further underscoring the phenotypic heterogeneity within CCS.

In our patient, malignant transformation to spiradenocarcinoma occurred in the context of biallelic CYLD inactivation together with homozygosity for a germline missense variant in *MSH2* (c.1609A > G; p.Lys537Glu), suggesting a cooperative genetic event beyond CYLD loss. CYLD truncation is sufficient to initiate benign adnexal tumorigenesis, but progression to malignancy typically requires additional drivers. Genomic studies of spiradenocarcinomas and cylindrocarcinomas have identified recurrent somatic mutations in *TP53*, *EP300*, *KDM6A*, *BCOR*, *DNMT3A*, *MBD4*, *ALPK1*, *MIB2*, and *PIK3R1*, affecting pathways such as cell cycle regulation, chromatin remodeling, and inflammatory signaling (9, 10, 12, 28). Although MMR deficiency has not been established as a recurrent driver for malignant transformation in adnexal tumors or cutaneous squamous cell carcinoma, previous whole-exome studies in spiradenocarcinomas identified mutational signatures of defective MMR with intact protein expression and MSS phenotype (9, 10, 29).

Our *in silico* analyses predict a deleterious effect of the *MSH2* variant, whereas IHC shows preserved expression. The variant lies within the highly conserved clamp domain, essential for DNA binding and heterodimer stabilization. Missense changes in this region may retain nuclear localization yet impair MMR function through defective mismatch recognition, destabilization of the MSH2–MSH6 heterodimer, or altered ATPase activity (30–32), providing a plausible explanation for the discordance between computational predictions and immunohistochemical findings. Nevertheless, considering that the AlphaMissense score (0.6059) lies in a moderate range and the variant has not yet been reported in ClinVar or functionally characterized, we deem it appropriate to maintain a conservative classification as a variant of uncertain significance. Although the family history does not meet Lynch syndrome criteria, the homozygous state of the variant and the presence of gastric cancer in a first-degree relative suggest a possible low-penetrance predisposition.

Taken together, these findings support a dual-driver model in which *CYLD* truncation initiates benign adnexal tumorigenesis, while compromised MSH2 function may facilitate malignant progression through reduced DNA repair fidelity and accumulation of secondary mutations. Functional validation, such as cell-based assays assessing the combined effects of CYLD loss and subtle MSH2 dysfunction, is necessary to confirm this proposed cooperative mechanism.

From a population genetics perspective, this case also highlights important implications for genomic testing in underrepresented groups. The patient’s genetic makeup was a mixture of Native American, European, and African ancestry (39.6, 42.2, and 18.2%, respectively). At the population level, Ecuadorians generally exhibit predominant Native American ancestry with variable African and European contributions (e.g., NAM ≈ 0.64, EUR ≈ 0.16, AFR ≈ 0.20) (33, 34). Although our patient displayed a higher proportion of European ancestry, her profile lies within the wide interindividual and regional variation reported in Ecuador, supporting the interpretation of variant rarity in an underrepresented population. Clinically, this highlights the importance of integrative genomic profiling in CCS with malignant transformation since standard IHC and microsatellite instability testing may fail to capture functionally relevant MMR variants. Exome sequencing combined with structural modeling provides added diagnostic value, and functional studies will be necessary to validate the impact of rare MMR alterations and to clarify the contribution of cryptic MMR dysfunction to malignant progression in adnexal tumors. Given the rarity of CCS-associated spiradenocarcinoma and the limited representation of Latin American genomes in reference databases, deposition of this variant into ClinVar would support future interpretation and enable comparison as additional cases are reported.

## 4 Conclusion

This case represents the first genetically confirmed report of CCS in Ecuador and one of the few described in South America, thereby expanding the geographic and clinical understanding of this rare disorder. The identification of a pathogenic *CYLD* nonsense variant together with a candidate *MSH2* modifier illustrates the diagnostic value of exome sequencing in patients with atypical or malignant presentations. Beyond its geographic novelty, this case underscores the importance of integrative molecular and structural analyses in clarifying cooperative mechanisms of malignant progression. Incorporating genomic tools into dermatologic evaluation will be essential in order to refine surveillance strategies, identify at-risk individuals, and strengthen genotype–phenotype correlations in adnexal tumor syndromes.

## Data Availability

The original contributions presented in the study are included in the article/Supplementary material, further inquiries can be directed to the corresponding author.

## Footnotes

1
https://www.ncbi.nlm.nih.gov/tools/primer-blast/

2
https://www.ebi.ac.uk/interpro/

3
https://smart.embl.de/
