# Supplementary material for: Case Report: CYLD cutaneous syndrome with malignant transformation to spiradenocarcinoma: cooperative effects of CYLD truncation and an MSH2 clamp-domain variant in an Ecuadorian patient
Source: Front Med (Lausanne). 2026 Feb 18;13:1703885. doi: 10.3389/fmed.2026.1703885 (PMC12956719; doi:10.3389/fmed.2026.1703885)
Supplement: Supplementary file 1 [file Table_1.docx]

CYLD Cutaneous Syndrome with Malignant Transformation to Spiradenocarcinoma: Cooperative Effects of CYLD Truncation and an MSH2 Clamp-Domain Variant in an Ecuadorian Patient

Carlos Reyes-Silva^1^, Gabriela Jaramillo-Koupermann^2^, Maritza Quishpe^3^, Rosa Pacheco^3^, Skehirly Burgos-Tapia^4^, Ana Karina Zambrano^5^, Alejandro Cabrera-Andrade^6*^

^1^Unidad de Genética, Hospital de Especialidades Eugenio Espejo, Quito, Ecuador

^2^Laboratorio de Biología Molecular, Subproceso de Anatomía Patológica, Hospital de Especialidades Eugenio Espejo, Quito, Ecuador

^3^Anatomía Patológica, Hospital de Especialidades Eugenio Espejo, Quito, Ecuador

^4^Escuela de Enfermería, Facultad de Ciencias de la Salud, Universidad de Las Américas, Quito, Ecuador

^5^Universidad UTE, Facultad de Ciencias de la Salud Eugenio Espejo, Centro de Investigación Genética y Genómica, Quito, Ecuador

^6^Grupo de Bio-Quimioinformática, Universidad de Las Américas, Quito, Ecuador

# Supplementary Tables

Supplementary Table S1. Primer sequences for amplification of variants in CYLD and MSH2.

| **Primer ID** | **5'- 3'** |
| --- | --- |
| **CYLD_F** | GGAGCGAGAACACTGTTGGA |
| **CYLD_R** | GGCGAAATCTGCACAAAACCT |
| **MSH2_F** | TTGGACCCTGGCAAACAGAT |
| **MSH2_R** | AAGCAGAGATATGAGGGGGAAC |

**Supplementary Table S2.** CYLD domain architecture and functional annotation.

|  |  |  |
| --- | --- | --- |
| **Domain / Region** | **Amino Acids (aa)** | **Function^1^** |
| USP domain | 598–947 | Catalytic domain; removes K63- and M1-linked polyubiquitin chains from NF-κB pathway components and other substrates. Essential for tumor suppressor activity. |
| B-box subdomain | 786–853 | Zinc-binding motif inserted in the USP domain; mediates protein–protein interactions and cytoplasmic localization. No intrinsic ubiquitin ligase activity. |
| CAP-Gly 1 | 127–203 | Microtubule association; supports microtubule assembly, cell migration, and possibly cell cycle regulation. |
| CAP-Gly 2 | 232–303 | Contributes to structural support and cytoskeletal interactions; less directly linked to NF-κB regulation. |
| CAP-Gly 3 / NEMO-binding site | 472–540 | Microtubule-binding SH3-like fold; required for NEMO binding, recruitment to the IKK complex, and suppression of NF-κB activation. |
| TRAF2 interaction site | 394–469 | Binds TRAF2, an adaptor protein in TNF receptor signaling; regulates NF-κB and MAPK pathways. Includes the PVQES motif (aa 453–457). |
| PVQES motif (within TRAF2 site) | 453–457 | TRAF2-binding motif; essential for direct association with TRAF2 and related adaptors. |
| Phosphorylation cluster (“phospho-patch”) | 418–444 | Serine-rich region phosphorylated in response to TNF-α, mitogens, and LPS; modulates CYLD enzymatic activity and protein–protein interactions dynamically. |
| Proline-rich motifs | 388–413, 446–471 | Facilitate interactions with SH3-domain proteins; support NEMO binding via CAP-Gly 3. |
| TRIP interaction region | 106–593 | Broad adaptor protein-binding range; contributes to protein interactions but less directly implicated in CCS-specific mechanisms. |

^1^Domain boundaries and functions were compiled from UniProt (Q9NQC7), InterPro, Pfam, and published functional studies (Bignell et al., 2000; Sun, 2010; Dubois, 2020).

## Supplementary Figures


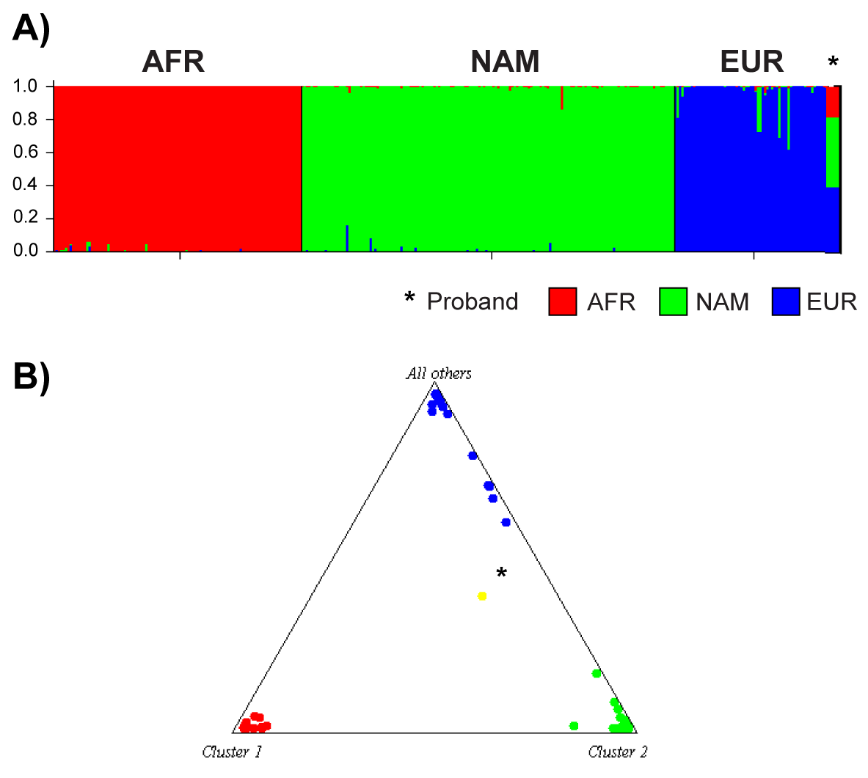


**Supplementary Figure S1.** Genetic ancestry analysis of the proband. **(A)** Ancestry proportions estimated using 46 ancestry-informative markers (AIMs), showing African (AFR), Native American (NAM), and European (EUR) contributions. The proband is indicated by an asterisk. **(B)** Ternary plot positioning the proband (asterisk) in relation to reference populations, illustrating the admixture profile.
